# Molecular and Pathological Detection of Hepatitis E Virus in Roe Deer (*Capreolus capreolus*) and Fallow Deer (*Dama dama*) in Central Italy

**DOI:** 10.3390/vetsci9030100

**Published:** 2022-02-24

**Authors:** Niccolò Fonti, Maria Irene Pacini, Mario Forzan, Francesca Parisi, Marcello Periccioli, Maurizio Mazzei, Alessandro Poli

**Affiliations:** 1 Dipartimento di Scienze Veterinarie, Università di Pisa, Viale delle Piagge, 2-56124 Pisa, Italy; niccolo.fonti@phd.unipi.it (N.F.); mariairene.pacini@phd.unipi.it (M.I.P.); mario.forzan@unipi.it (M.F.); francesca.parisi@vet.unipi.it (F.P.); maurizio.mazzei@unipi.it (M.M.); 2 Unità Funzionale di Sanità Pubblica Veterinaria e Sicurezza Alimentare Zona Distretto Grossetana, Dipartimento di Prevenzione, Azienda USL Toscana Sud Est, Amiata Grossetana e Colline Metallifere, Viale Cimabue, 109-58100 Grosseto, Italy; marcello.periccioli@uslsudest.toscana.it

**Keywords:** deer, ELISA, HEV, immunohistochemistry, inflammatory infiltrates, liver pathology, PCR, zoonosis, wildlife

## Abstract

Hepatitis E virus (HEV) is a common causative agent of acute hepatitis in the world, with a serious public health burden in both developing and industrialized countries. Cervids, along with wild boars and lagomorphs, are the main wild hosts of HEV in Europe and constitute a documented source of infection for humans. The aim of this study was to evaluate the presence of HEV in roe deer (*Capreolus capreolus*) and fallow deer (*Dama dama*) living in Tuscany, Central Italy. Liver samples from 48 roe deer and 60 fallow deer were collected from carcasses during the hunting seasons. Following the results obtained from molecular and histopathologic studies, 5/48 (10.4%) roe deer and 1/60 (1.7%) fallow deer liver samples were positive for the presence of HEV RNA. All PCR-positive livers were also IHC-positive for viral antigen presence, associated with degenerative and inflammatory lesions with predominantly CD3+ cellular infiltrates. This study represents the first identification in Italy of HEV RNA in roe and fallow deer and the first study in literature describing liver alterations associated with HEV infection in cervids. These results demonstrate that HEV is present in wild cervid populations in Italy and confirm the potential zoonotic role of these species.

## 1. Introduction

Hepatitis E virus (HEV) is the leading cause of an acute viral hepatitis, namely hepatitis E, causing serious public health burden in both developing and industrialized countries around the world [1]. HEV belongs to the *Hepeviridae* family, including 2 genera: *Orthohepevirus* (comprising 4 species: *A*, *B*, *C*, and *D*) and the monospecific *Piscihepevirus* genus [2]. *Orthohepevirus A* is the most studied species and despite representing a single serotype it is divided into at least eight genotypes (HEV1-8). Four major genotypes (HEV1–4) are plainly implicated in human infection. Genotypes 1 and 2 (HEV1-2) are strictly human viruses and present epidemic forms via a fecal–oral route in developing countries (Africa, Asia, and Latin America), with outbreaks mainly due to water contamination. On the other hand, genotypes 3 and 4 (HEV3-4) have a wide host range including multiple different mammals (such as ungulates, lagomorph, rodents, and humans) and cause sporadic zoonotic cases primarily as a foodborne pathogen both in industrialized and developing countries.

Clinically, HEV causes an acute self-limiting hepatitis (2–6 weeks), but in most individuals (both animals and humans) infection often runs asymptomatic or causes only mild systemic disease. Though the mortality rate is low, genotypes 1 and 2 could be responsible for severe manifestations especially in weakened hosts such as pregnant women and immunocompromised people, while zoonotic genotypes (HEV3-4) are associated with chronic development [3]. In Europe, as for others developed countries, autochthonous cases due to HEV3 infection are increasing and exceeding the number of imported cases due to the introduction of HEV1-2 [4]. Though genotypes 3 is divided into multiple subtypes (at least 13) grouped into 3 main clades [5], the classification is very dynamic and there are many emerging and not yet classified strains, such as the 3l and 3n strain [6]. Recently, the circulation of subtypes 3e and 3f, but also 3a, 3c, and 3l was reported in Italy, in both humans and animals [7].

Although domestic swine represent the HEV major reservoir, the occurrence of numerous HEV strains in wildlife and other domestic animals suggests their role as potential reservoirs and shows how HEV epidemiology is still partially understood [4]. Foodborne infections caused by the consumption of game meat or liver, eaten raw or uncooked, have increased [8]. In Italy, most of these cases have been diagnosed in the Abruzzo, Tuscany, Marche, and Lazio regions, where the consumption of traditional charcuterie products and extensive farming of native breeds and game are growing strongly [9].

The most important wild reservoir is certainly wild boar [10], but other ungulates from around the world are considered interesting in the role they could play in the epidemiology of hepatitis E [11]. It is remarkable that the first study that demonstrated the zoonotic potential of HEV was relative to the ingestion of raw meat of sika deer (*Cervus nippon*) by four Japanese citizens [12]. Following this evidence, the number of serological and molecular HEV surveys involving wild ruminants has increased exponentially but, until now, no studies have described the pathological changes induced by the virus in these wild hosts.

Because fallow deer (*Dama dama*) and roe deer (*Capreolus capreolus*) are widely distributed in Central Italy and represent, together with red deer (*Cervus elaphus*), the main species of cervids, the purpose of this study was to investigate HEV presence in fallow deer and roe deer in this area and to describe the histopathologic lesions associated with the viral infection.

## 2. Materials and Methods

### 2.1. Sample Estimation

**Figure 1:**
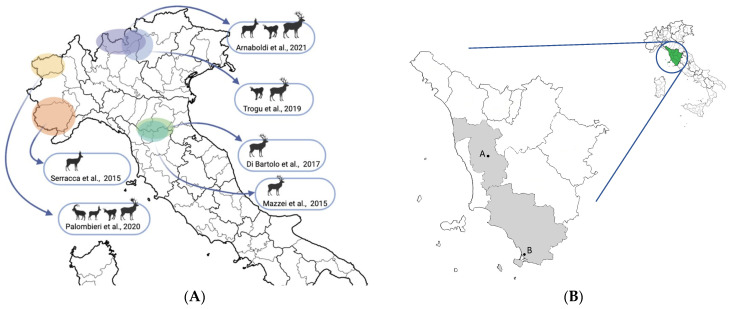
(**A**) Map of Northern and Central Italy with the geographical areas in which previous studies were conducted. No studies are reported in deer populations from Southern Italy. The references and the species included in each study are provided [22,24,25,26,27,28]. Created with BioRender.com (accessed on 10 February 2022); (**B**) Map of Tuscany with the two sampled areas in this study (Pisa and Grosseto provinces). A = Lajatico (PI), B = Orbetello (GR).

The sample size necessary to investigate the presence of HEV in the wild population was determined using the equation described by Cannon and Roe [13] with a confidence level of 95% considering three variables: the expected prevalence of HEV in the hosts; the consistence of free-ranging deer in the study area; and the reliability required of the conclusions. Previous serological studies conducted on cervids in many European countries assessed a seroprevalence between 0 and 13.9%, while HEV RNA was detected with a prevalence up to 15.4% in red deer, 34.4% in roe deer, and 4.4% in fallow deer in several European countries [14,15,16,17,18,19,20,21]. As for Italy (Figure 1A), an 11.0% prevalence of HEV RNA was highlighted in red deer from the province of Pistoia (Tuscany, Italy) [22]. Based on these data, an expected virological prevalence of 10%, with a confidence of 95% and a precision required of 10% were settled. As for free-ranging deer population, the consistency of roe and fallow deer in Tuscany was statistically proximal to an infinite population [23], with 30 thousand and 6 thousand animals shot per year in 2021, respectively (data published by Regione Toscana, www.regione.toscana.it, accessed on 10 February 2022). Hence, the minimum proper sample size resulted in 28 units for each species. Sample size was much less than the 5% of entire population, and no adjustment was needed.

### 2.2. Sample Collection

From November 2019 to April 2021, during the regular hunting seasons, samples from roe deer and fallow deer were collected in the provinces of Pisa (PI) and Grosseto (GR) (Tuscany, Italy) (Figure 1B). The sampling involved animals killed in accordance with the regional hunting regulations (Regolamento di attuazione della legge regionale 12 gennaio 1994 n°3 DPGR 48/R/2017) and transferred to two game-meat storing and processing establishments, respectively in Lajatico (PI) and Orbetello (GR).

Once the carcasses arrived at the establishments, during the slaughter procedures, samples from the liver, spleen, and mesenteric and retropharyngeal lymph nodes were collected for each subject. Representative samples of liver, spleen, and mesenteric and retropharyngeal lymph nodes tissue were fixed in a 10% buffered formalin solution (pH 7.4) and stored at room temperature for histopathological study, while one portion of liver was placed in sterile bags and stored immediately at −20 °C until virological investigations. All samples were transferred to the Department of Veterinary Science (Pisa, Italy).

A total of 108 animals was included in this study: 48 roe deer (13 from the Pisa and 35 from the Grosseto province) and 60 fallow deer (8 from the Pisa and 52 from the Grosseto province). Each subject was classified according to sex, hunting area, age (0–1 year fawn; 1–2 years yearling; >2 years mature), and sampling data. Age was estimated from the body mass, the morphological characteristics, the dental eruption, and wear of the lower molar arch [29].

### 2.3. ELISA Screening

All liver samples were subjected to a virological screening, to select the samples on which to proceed with the molecular investigation. Following the indications by the ELISA Sample Preparation Guide (Abcam, Cambridge, UK), 10 mg of tissue were dipped in 600 μL of lysis buffer (1% Triton X-100) and, after 2 h incubation at 4 °C with constant stirring, the extract was centrifuged at 13,000× *g* rpm for 5 min and the supernatant was stored at −80 °C.

Enzyme immunoassay screening was performed with the commercial kit HEPATITIS E–HEV-Ag (XpressBio, Frederick, MD, USA), a double antibody “sandwich” ELISA assay for antigen detection following manufacturer’s instructions. Three negative controls (NC), two positive controls (PC), and a blank (BLK) were used, while 100 μL of tissue extract was loaded into the remaining wells. In addition to the controls supplied with the kit, tissue extracts obtained from two negative and two positive rabbit liver identified in a previous study [30] were used as internal controls. The colorimetric reaction was stopped after 30 min of incubation in the dark at 37 °C by adding 50 μL of sulfuric acid-based stop solution, and the optical density (OD) of each well was acquired by spectrophotometric reading at 450 nm (Multiskan FC; Thermo Fisher Scientific, Waltham, MA, USA). Following the manufacturer’s indication, the results were interpreted by relating the OD of each sample (Sn) with a cut-off (CO) obtained as the OD mean of the NC plus 0.12. Samples with a ratio Sn/CO <1 were considered as negative, and those with a ratio Sn/CO >1 were considered as positive. Since the ELISA test was performed as a screening, doubtful samples were also considered as positive, to increase the sensitivity of the test.

### 2.4. Molecular Analysis

Antigen ELISA-positive liver tissue samples underwent molecular investigations, by a first TaqMan real-time reverse-transcription PCR (RT-qPCR) assay, followed by an end-point RT-PCR.

PCR reactions were preceded by the extraction of the total RNA from tissue samples using the RNeasy mini kit column extraction system (Qiagen, Hilden, Germany). According to manufacturer’s instruction, 30 mg of liver from each sample were soaked in 600 μL of lysis buffer (RLT) and disrupted and homogenized by TissueLyser II (Qiagen, Hilden, Germany) at the beginning of the procedure. All the obtained RNAs were quantified using NanoDrop (Thermo Fisher Scientific, Waltham, MA, USA).

To achieve an absolute quantification of the viral RNA present in the samples, a calibration standard curve was generated. A 296-base pairs (bps) synthetic oligonucleotide comprising the ORF3 target fragment of the RT-qPCR was cloned into the pcDNA3.1 (+) plasmid vector under the region containing the T7 polymerase promoter (GenScript Biotech, Leiden, Netherlands). The plasmid was linearized by digestion with Hind III (NEB, Ipswich, MA, USA) in a restriction site located downstream from the target sequence and purified by MiniElute Reaction Cleanup Kit (Qiagen, Hilden, Germany). The linearized DNA was then transcribed in vitro using the MAXIscript SP6/T7 kit (Thermo Fisher Scientific, Waltham, MA, USA) according to the manufacturer’s indications and the RNA was purified using the MiniElute RNeasy Cleanup Kit (Qiagen, Hilden, Germany). The quantity of RNA was estimated by NanoDrop (Thermo Fisher Scientific, Waltham, MA, USA) and serially diluted from 4 × 10^6^ to 4 × 10^1^ molecules of RNA/μL.

In this study, 2 μL of RNA was analyzed using the Luna Universal Probe One-Step RT-qPCR Kit (NEB, Ipswich, MA, USA), with a set of primers and probes previously described by Jothikumar et al. [31] and directed towards a highly conserved 69-bps fragment of ORF3. HEV RNA extracted from infected wild boar liver used in a previous study was used as an internal positive control (IPC) [9]. The viral load was reported as viral copies number/100 ng of RNA. On the samples that resulted positive at RT-qPCR, a conventional end-point RT-PCR assay was performed, using the same forward and reverse real-time primers, in order to obtain amplicons for sequencing. The reaction was carried out with the commercial OneStep RT-PCR Kit (Qiagen, Hilden, Germany) following the instructions provided by the manufacturer and the PCR products were identified by electrophoretic run on 1.5% TBE agarose gel.

In order to confirm the specificity of the RT-qPCR, sanger sequencing was conducted at the BMR Genomics company (Padua, Italy) on the amplicons obtained by RT-PCR. The sequences obtained were then compared with other HEV sequences present on GeneBank using the online software BLAST (Basic Local Alignment Search Tool) and the software BioEdit 7.2 as biological sequence alignment editor [32].

### 2.5. Histopathology and Immunohistochemistry

Four-micrometer-thick serial sections from formalin-fixed paraffin-embedded liver, spleen and lymph node tissue samples were stained with H&E for histopathological examination. For immunohistochemistry (IHC), other serial sections of the different organs were cut, mounted on polarized slides Menzel–Gläser Superfrost plus (Thermo Fisher Scientific, Waltham, MA, USA), dewaxed in xylene for 6 min, and rehydrated through decreasing graded alcohols (100 and 95%) and water. Antigen retrieval was achieved placing the slides in 10 mM of sodium citrate (pH 6), 10 mM of Tris base, or in 1 mM of EDTA solution (pH9), depending on the primary antibody (Table 1), and boiling for 15 min in an 800-W microwave oven. The slides were then cooled down at room temperature, washed with running tap water, and mounted on Shandon Coverplate (Thermo Fisher Scientific, Waltham, MA, USA). Peroxidase and protein block were performed as reported in Table 1. A mouse monoclonal antibody anti-HEV clone 4B2 (Chemicon International, Temecula, CA, USA), successfully used in a previous study on rabbits [30], was used to immunolocalize HEV antigen in liver samples with a 1:200 dilution in a buffer solution (PBS) and an overnight incubation at room temperature. In addition, the leucocytic infiltration in the livers was phenotyped with an in-house optimized leukocyte immunophenotyping protocol. Specific IHC was conducted with an anti-human CD3 rabbit polyclonal antibody (Dako, Glostrup, Denmark) diluted 1:200 directed against T-lymphocytes; with an anti-human CD20 rabbit polyclonal antibody diluted 1:100 (Thermo Fisher Scientific, Rockford, IL, USA) directed against the B-lymphocytes; with an anti-Iba-1 rabbit polyclonal antibody diluted 1:300 for the detection of macrophages. The specific protocol and dilution for each antigen is presented in Table 1.

A biotinylated goat anti-mouse IgG polyclonal antibody (Vector Laboratories, Burlingame, CA, USA) for HEV sections and a biotinylated horse anti-mouse/rabbit IgG polyclonal antibody (Vector Laboratories, Burlingame, CA, USA) for leukocytes markers, were used as secondary antibodies with 30 min incubation at room temperature. Binding was detected by a streptavidin-peroxidase kit (R.T.U. Horseradish Peroxidase Streptavidin, Vector Laboratories, Burlingame, CA, USA) and the colorimetric reaction was developed by incubating 3-1-diaminobenzydine as a substrate (ImmPACT DAB Peroxidase Substrate kit, Vector Laboratories, Burlingame, CA, USA). Slides were then counterstained in hematoxylin for 50 s, dehydrated in increasing graded alcohols (95 and 100%) and then cleared with xylene. Sections were mounted using DPX (Leica Microsystems, Wetzlar, Germany) and observed under light microscope. The liver of a PCR-positive and IHC-positive rabbit was used as a positive control. As a negative control, the primary antibody was replaced with an irrelevant, isotype-matched antibody to control for non-specific binding of the secondary antibody.

### 2.6. Statistic Analysis

The Fisher’s exact test was used to compare the differences between the prevalence values in the different subsets of the population tested and evaluate their significance. A threshold value of statistical significance *p* < 0.05 was chosen and the variables taken into consideration were sex, age, province of origin, and seasonality. For the latter factor, the samples were divided into two dichotomous variables based on the date of death (periods March–August and September–February).

## 3. Results

### 3.1. Animals

The roe deer samples were 17/48 males (35.4%) and 31/48 females (64.6%), while the fallow deer samples were 38/60 males (63.3%) and 22/60 females (36.7%). As regarding the age, 43/108 subjects were classified as yearling (39.8%) and 49/108 were classified as matures (45.4%), while 16/108 (14.8%) subjects were of undetermined age (Table 2). When shot, all the deer appeared asymptomatic.

### 3.2. Virological Investigation

As result of immunoenzymatic investigation, a total of 19/108 (17.6; 95% CI: 11.6–25.9) samples were positive: 16 of them belonged to roe deer (16/48; 33.3%; 95% CI: 21.7–47.5) and three (3/60; 5%; 95% CI: 1.7–13.7) to fallow deer. Almost all the ELISA-positive roe deer specimens belonged from Grosseto province (15/16) and 1/16 from Pisa province, while all the fallow deer ELISA-positive samples came from Grosseto (3/3).

The spectrophotometric analyses conducted on the RNA extracted from the 19 ELISA-positive liver tissue samples showed that all the samples were suitable for subsequent molecular investigations with a concentration of RNA with a mean of 536 ± 446 ng/μL. RT-qPCR identified 6/19 (31.6%) positive liver samples, 5/6 belonging to roe deer and 1/6 to a fallow deer (Table 3). Amplification of positive samples was observed from 20 to 25 cycles.

Based on the results of RT-qPCR, the total prevalence in tested deer was 5.5% (95% CI: 2.6–11.6) (6/108), with a prevalence of 10.4% (95% CI: 4.5–22.2) (5/48) in roe deer and 1.7% (95% CI: 0.3–8.9) (1/60) in fallow deer. Following end point PCR protocol, good quality bands with a height (of 69 bps) in accordance with what expected were obtained and one of them (sample #5) was considered suitable for sequencing.

The analysis of the nucleotide sequence revealed an identity of 98% (39/40) with the 5288 to 5327 portions of the genome of an *Orthohepevirus A* strain, genotype 3e isolated in Italy from wild boar liver and deposited in GenBank (Acc. No. MT840367.1) by Aprea et al. [33] with an Expect (E) value of 1 × 10^−12^, confirming the specificity of the molecular analysis.

The results of immunoenzymatic and molecular analyses are summarized in Table 4.

### 3.3. Histopathologic and Immunohistochemical Investigations

**Figure 2:**
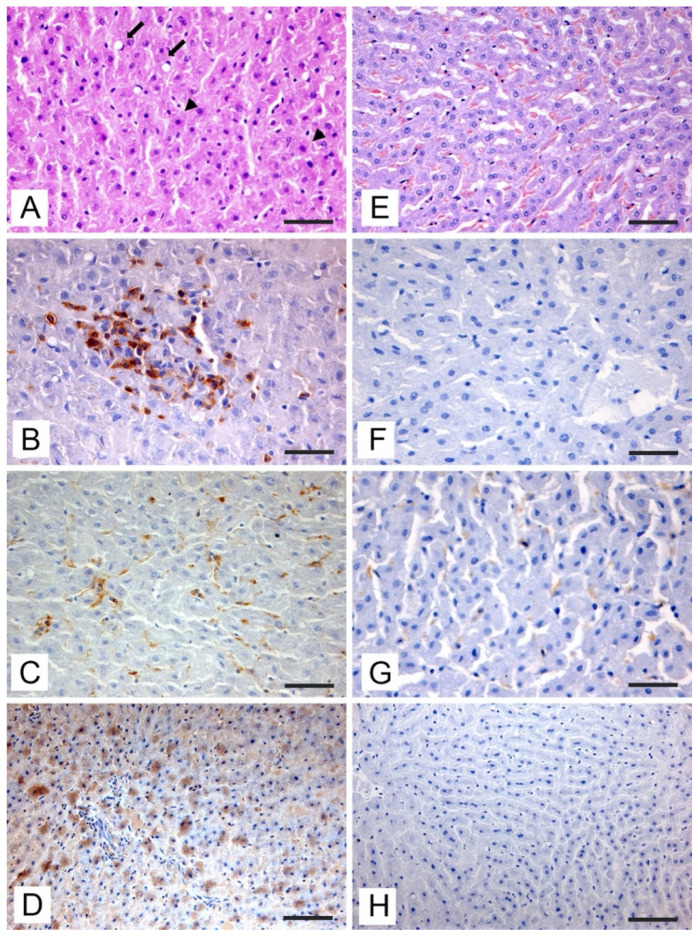
Liver tissue sections (pericentrilobular areas) from PCR-positive roe deer (**A**–**D**) and PCR-negative subject (**E**–**H**). (**A**) Focal area of degeneration with Ito cell hyperplasia (arrow) and single hepatocytes with nucleus loss and cytoplasmic hypereosinophilia (arrowheads) (**H**,**E**, bar = 50 μm). (**B**) CD3+ inflammatory infiltrate associated with degenerating hepatocytes (IHC, bar = 50 μm). (**C**) Hyperplasia of Kupffer cells labeled with anti-Iba-1 (IHC, bar = 50 μm). (**D**) Immunohistochemical staining with cytoplasmic pattern of hepatocytes incubated with anti-HEV antibody (IHC, bar = 100 μm). (**E**) Normal hepatocytes in the pericentrilobular area (**H**,**E**, bar = 50 μm). (**F**) Absence of CD3+ lymphocytic infiltrate in hepatic sinusoids (IHC, bar = 50 μm). (**G**) Scattered Kupffer cells labeled with anti-Iba-1 antibody (IHC, bar = 50 μm). (**H**) Absence of immunostaining after incubation with anti-HEV antibody (IHC, bar = 100 μm).

**Figure 3:**
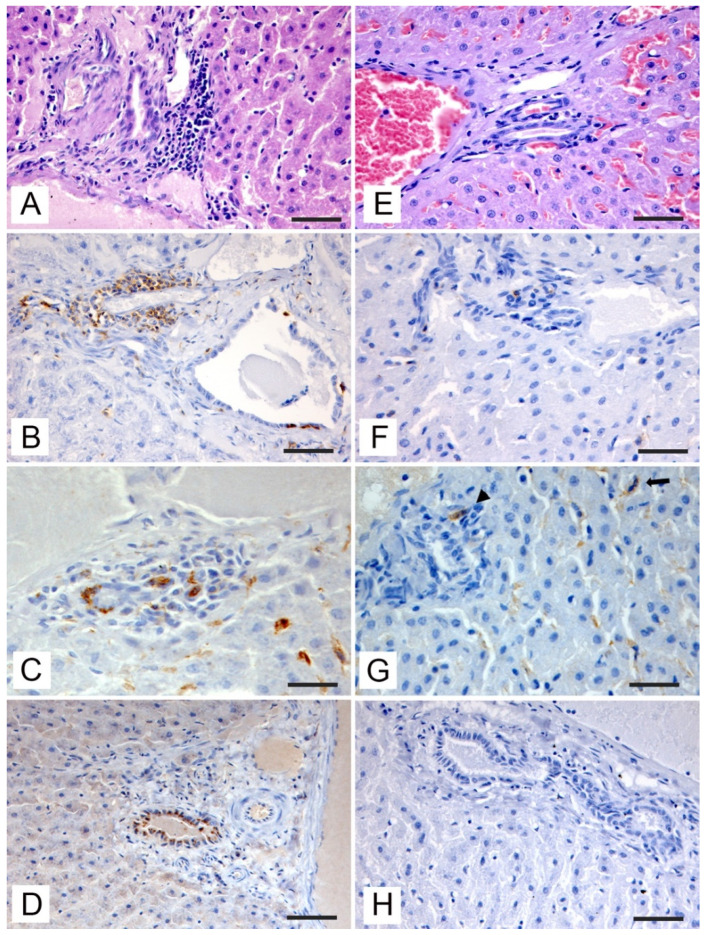
Liver tissue sections from PCR investigated samples. (**A**) HEV-positive roe deer; periductal lymphocytic infiltration in the portal space (**H**,**E**, bar = 50 μm). (**B**) HEV-positive roe deer; CD3+ periductal inflammatory infiltrate with small clusters of cells in contact with the basal dominium of positive cholangiocytes (IHC, bar = 100 μm). (**C**) HEV-positive roe deer; cluster of macrophages infiltrating the portal space stained with anti-Iba-1 antibody (IHC, bar = 50 μm). (**D**) HEV-positive roe deer; immunohistochemical staining with granular cytoplasmic pattern in cholangiocytes incubated with anti-HEV antibody (IHC, bar = 100 μm). (**E**) Roe deer HEV-negative; absence of periductal inflammatory infiltrate (**H**,**E**, bar = 50 μm). (**F**) HEV-negative roe deer; little amount of CD3+ periductal lymphocytes (IHC, bar = 50 μm). (**G**) HEV-negative roe deer; resident Kupffer cells labeled with anti-Iba-1 antibody (arrow) and a single macrophage cell, also labeled (arrowhead) (IHC, bar = 50 μm). (**H**) HEV-negative roe deer; cholangiocytes with no staining after incubation with anti-HEV antibody (IHC, bar = 100 μm).

During post-mortem examination, none of the subjects included in the study showed gross lesions or aspects attributable to pathological states. Histopathologic changes observed in the liver of RT-qPCR-positive roe and fallow deer are presented in Figure 2 (pericentrilobular areas) and in Figure 3 (periportal areas).

The histopathological investigation highlighted the presence of liver changes both in the pericentrilobular and periportal areas. In the pericentrilobular areas, a mild hepatitis characterized by lymphocytic infiltration and hyperplasia of Kupffer cells with small focal cluster of which showed pycnotic nuclei and cytoplasmic hypereosinophilia, attributable to an initial stage of apoptosis, was evident (Figure 2A). The inflammatory infiltrates were composed of a prevalence (over 85%) of small CD3+ T-lymphocytes (Figure 2B), associated with significant hyperplasia of the Kupffer (Figure 2C). Viral antigen with a distinct cytoplasmic staining was evident in the hepatocytes (Figure 2D). Immunostained hepatocytes were located in the areas where apoptosis was observed.

In the portal spaces, at the periductal level a mild infiltration of lympho-mononuclear cells was evident (Figure 3A). Up to 85% of the inflammatory cells were CD3+ T-lymphocytes (Figure 3B). There were also clusters of intensely stained Iba-1-positive infiltrating macrophages (Figure 3C). Small scattered CD20-positive B-lymphocytes (up to 5% of the infiltrate) were present, the number of these cells was significantly reduced compared to CD3-positive T-lymphocytes. The viral antigen was immunolocalized in the cholangiocytes of the biliary epithelium with a slightly granular pattern (Figure 3D).

Of note, the immunohistochemical results of HEV antigen staining showed concordance with RT-qPCR both in roe deer and fallow deer. All the PCR-positive samples showed positive staining when incubating with the anti-HEV antibody, while PCR-negative subjects did not show any immunostaining, either at the hepatocyte level (Figure 2H), or at the ductal level (Figure 3H) with a significantly reduced presence of inflammatory cells in the pericentrilobular and periportal areas (Figure 2E and Figure 3E) and a minimum number of CD3-positive T-lymphocytes (Figure 2F and Figure 3F) and macrophages (Figure 2G and Figure 3G).

The hepatic lesions described were present in a discontinuous way in the examined samples and the distribution of these alterations is presented in Table 5. The exam of extrahepatic tissues (spleen and lymph nodes) of HEV-PCR-positive deer did not highlight nor histologic alterations (e.g., inflammatory infiltrates) nor HEV antigen when incubating with the anti-HEV antibody.

### 3.4. Statistical Analysis

Regarding the prevalence highlighted, no significant differences were found about age and origin of the samples of both roe and fallow deer, while male roe deer showed a higher risk of infection than female subjects (*p* = 0.0184) and all the positive samples of roe deer were shot in the March–August period (*p* = 0.0085).

## 4. Discussion

In this study, total cervids HEV-RNA positivity was 5.5% (6/108), with a prevalence in roe and fallow deer of 10.4% (5/48) and of 1.7% (1/60), respectively. These values are in line with the 10% prevalence previously reported in a red deer population from the Tuscan-Emilian Apennines [22] and in accordance with prevalence described in the rest of Europe for these species [11,21].

The importance of wild ruminants as potential HEV reservoirs and their zoonotic role is still under investigated [4]. Previous European studies have focused mainly on red and roe deer, often as an addition to wild boar investigations. Nevertheless, other species such as fallow deer and wild bovids have been included in some studies too. Generally, the European seroprevalence for these species is 0.0–13.9%, while the HEV RNA prevalence varies from 0 to 34.4% [11,34,35]. Few phylogenetically analyzable sequences have been isolated [36], which have shown HEV3 (subtypes 3a, 3e, 3f, and 3i related to wild boars and human infection) as the only circulating genotype in European cervids, while HEV4 has been found in Asia [11,21,37]. The first study demonstrating HEV presence in roe deer populations in Europe was conducted in Hungary. In this study, 11/32 (34.4%) roe deer liver samples harbored HEV3 RNA [34]; 3e and 3a subtypes, closely related to domestic pig strains, were subsequently identified in this species [14]. Roe deer HEV infection was described also in Czech Republic (1/30; 3.33%) [17], Germany (5/78; 6.4%) [19], and Lithuania (21/93; 22.58%) [21]. Regarding fallow deer, a 4.3% prevalence (2/46) was described in Germany [18].

In Italy, the first HEV investigation on cervids was carried out in 2015 by testing 30 samples of roe deer liver from the province of Cuneo (Northern Italy) using an RT-qPCR assay; all samples scored negative [24]. The same year in Central Italy reported a seroprevalence in red deer of 5.60% (3/54) [25]. In the Tuscan-Emilian Apennines, Di Bartolo et al. [22] found a 13.9% seroprevalence in red deer, and HEV RNA was isolated from 11% of the sera tested and subtype 3e was identified. This study represented the first HEV isolation in cervids in Italy. The most recent studies were carried out in Northern Italy. A low seroprevalence has been shown in red deer and chamois (*Rupicapra r. rupicapra*) from the Alps [26]. In the Aosta Valley, sera and fecal samples from different wild ruminants were tested: 2.6% of red deer, 3.1% of roe deer, and 6.3% of ibex (*Capra ibex*) were seropositive, while no fecal sample was positive for RT-PCR. According to the authors this was the first report of HEV seropositivity in Italy for both roe deer and ibex [27]. A further molecular investigation on 218 red deer, 6 roe deer, and 4 chamois from the province of Sondrio, revealed no cervids positive for HEV [28]. Recently, in a serological survey performed in wild ungulates living in Alpine and pre-Alpine districts of the province of Bergamo seropositive subjects were detected in chamois (5/92; 5.1%) roe deer (1/227; 0.4%) and mouflons (*Ovis musimon*) (1/49; 2%) populations [38].

In this paper, the quantification of the viral load showed a value of viral copies per 100 ng total RNA comparable with what has previously been reported, with the same analytical method, in wild boar liver samples [9]. This suggested that further studies are needed to assess whether the viral load reached in infected roe and fallow deer allows the elimination of infecting loads and intraspecific HEV circulation.

The use of serial diagnostic tests with a screening ELISA “high-throughput” test, allowed a greater sample size, essential due to the discontinuous epidemiological pattern of HEV, and the optimization of overall specificity, positive predictive value (PPV) and costs [4]. The HEV pORF2 antigenic enzyme immunoassay kit used in this paper was biologically independent with respect to common molecular investigations. Serology was not chosen as a screening test due to: (I) the excessive degree of hemolysis in the blood samples of deer carcasses delivered to the slaughterhouse; and (II) the reduced concordance between seropositivity and simultaneous HEV infection reported in previous studies [19,30,39]. This phenomenon is not clearly understood, but it could be due to an acute and self-limiting trend HEV infection, with complete viral clearance as described in other sensitive species [40,41].

Nucleotide sequencing confirmed results obtained from molecular analysis and identified the HEV3 origin of the amplicon. Due to the small size of the genomic fragment, similarly to other studies conducted on these wild species [15,17,18], it was not possible to carry out a phylogenetic analysis of the strains involved. However, since the aim of this study is to detect the presence of HEV in the studied cervids population, sequencing analysis were carried out in order to verify HEV presence.

As regards to the histopathological investigation, no previous studies in cervids are known [42]. The present study represents the first report that describes the histological changes associated with HEV infection in both roe and fallow deer. A mild lymphocytic colangiohepatitis, in the absence of clinical symptoms and macroscopic alterations, was described in other species following natural and experimental infections [43]. Immunostaining highlighting the presence of viral antigens was evident both in hepatocytes in the hepatic parenchyma and in the epithelial cells of the small bile ducts [40]. The characterization of the lymphocyte infiltrates associated with HEV+ immunostained cells allowed to observe a clear prevalence of T-lymphocytes compared to B-lymphocytes [44]. Moderate hyperplasia of Kupffer cells in response to viral infection was also described in all PCR positive samples. These resident cells, with their activity as “scavengers” of the liver tissue, may play multiple roles. Indeed, they can represent a source of chemotactic molecules for T-lymphocytes and induce the myofibroblast transition into Ito cells by promoting fibrosis [45]. Unlike other studies, no necrotic-degenerative lesions were found, and the pathological changes were milder than those found in other species susceptible to infection [43,46,47]. Since HEV seems not to be a cytopathogenic virus, the viral induced liver damage was predominantly mediated by the host inflammatory cells [3]. In this perspective it would be useful to develop additional immunohistochemical markers for cervids immune cells immunophenotyping in order to further characterize involved leukocyte subpopulations (e.g., CD8+, CD4+, NK, T reg, Th17, and plasma cells) and acquire additional information on the pathogenetic mechanisms of HEV. Immunohistochemical investigation results were in agreement with molecular studies, thus emerging as a valid tool for epidemiological investigations, allowing the association of histological changes to PCR analysis and discriminating real infection from cross contamination of the sample.

In this paper, the prevalence of HEV infection in roe deer (10.4%) was found to be comparable to wild boars and rabbits from the same geographical areas, respectively 12% and 9% [9,30], while the prevalence in fallow deer was lower, with only one subject of 60 (1.7%). Further studies are needed to understand the dynamics of HEV diffusion between different species within the same environment. The risks factors, the interrelationships between humans, suidae, deer, lagomorphs, and carnivores must still be fully understood [25,48].

In Europe, cervids show reduced average infection rates and seroprevalences compared to wild boar from the same areas [49] and it has also been shown that liver viral loads found in infected deer are lower than in wild boar [19]. These data suggest that deer could not be true HEV reservoirs, but that they probably represent secondary hosts who are accidentally infected by sharing the same habitat. Regarding cross-specific transmission, there is no experimental evidence of the sensitivity of cervids to infection with HEV strains originating from other animals, but identical HEV sequences in wild boar, cervids, and other animal species to the same geographical areas are a common finding [16,49], suggesting that interspecific transmission of HEV occurs in nature. In this study the highest nucleotide similarity (98%) was found in a wild boar isolated strain in Abruzzo (Acc. No. MT840367.1) and further HEV3 strains isolated from human patients, too. However, the length of the sequenced amplicon was too short to allow further analysis.

Interspecific aggregation around feeding sites could enhance HEV transmission, especially in those periods of the year when trophic resources are reduced. In support of this, in Hungary all HEV-positive wild boar and deer were collected between February and April [14]. Similarly, in this study all roe deer positive samples were collected in the spring period (*p* = 0.0085). Moreover, the prevalence was higher in males than in females (*p* = 0.0184), this phenomenon was also reported by Di Bartolo and colleagues [22] and it is conceivable that the greater mobility on the territory typical of male roe deer in the late winter period could enhance the chance to meet other species sensitive to infection [50]. Furthermore, the roe deer extreme adaptability to different environments, especially in ecotonal areas where the probability of contact with both domestic and wild animals is greater [49], could explain the higher prevalence found in this species.

Seroprevalence found in cervids in Scandinavian countries, where wild boar is almost absent [35] and the evidence of zoonotic role played by rabbits and rodents [51] suggest that the wild boar is not the only reservoir of HEV.

In this study, both the prevalence of roe deer and the viral load in the liver tissue of infected subjects were not lower than those highlighted in previous studies on wild boars in the same area [9,28,52]. Furthermore, a recent paper suggests that the presence of deer populations is itself a risk factor for the spread of HEV infection in various wild boar populations [53]. All these elements are strongly indicative that cervids are not only dead-end hosts, but that they could contribute to keeping the hepatitis E virus in a specific territory, as well as representing a documented foodborne source of transmission for the man [12,54].

## 5. Conclusions

This paper represents the first identification of HEV RNA in roe and fallow deer liver tissues in Italy, indicating the presence of HEV in wild ungulates in Tuscany and the potential epidemiologic role of cervids. Moreover, this is the only study on cervids in which HEV-induced lesions and tissue immune response have been characterized, associating the description of the histopathological alterations and the immunohistochemical stain to the virological positivity. The reported zoonotic potential and the growing concern in consumption of game meat, could have an important role on the Public Health. For this reason, further studies are therefore necessary to gain more information on the risk associated with the consumption of meat from roe and fallow deer and to characterize HEV pathogenesis and epidemiology in these species.

## Figures and Tables

**Table 1:** Antibodies, reagents used for antigenic unmasking, blockade of endogenous enzymes and non-specific protein bonds, dilution of the primary antibody, and type of secondary antibody used.

Antibody	Ag Retrieval	Peroxidase Block	Protein Block	Dilution	II Ab
Anti-HEV	Tris-EDTA (pH 9)	H_2_O_2_ 3%	UV	1:200	Goat anti-M
Anti-CD3	Citric acid (pH 6)	BLOXALL B.S.	UV	1:200	Horse anti-M/R
Anti-CD20	Citric acid (pH 6)	BLOXALL B.S.	UV	1:100	Horse anti-M/R
Anti-Iba-1	Citric acid (pH 6)	BLOXALL B.S.	UV	1:300	Horse anti-M/R

**Table 2:** Number of animals sampled in this study sorted by species, province of origin, sex, and age. N.d. = not determined.

Species	Age	Pisa			Grosseto			Total
		Male	Female	Total	Male	Female	Total	
Roe deer	Yearlings (1–2 years)	1	2	3	6	5	11	14
	Mature (>2 years)	-	9	9	9	10	19	28
	Age n.d.	-	1	1	1	4	5	6
	Total	1	12	13	16	19	35	48
Fallow deer	Yearlings (1–2 years)	1	2	3	15	11	26	29
	Mature (>2 years)	3	1	4	12	5	17	21
	Age n.d.	-	1	1	7	2	9	10
	Total	4	4	8	34	18	52	60
Total cervids		5	16	21	50	37	87	108

**Table 3:** Sex, age class, date of sampling, hunting area, and viral load (viral copies/100ng of RNA) of PCR-positive subjects.

Species	N°	Sex	Age Class	Sampling Date	Hunting Area	PCR Viral Load
Roe deer	#1	F	yearling	04/03/21	Magliano in Toscana (GR)	1.40 × 10^3^
	#2	M	yearling	14/04/21	Scansano (GR)	2.03 × 10^4^
	#3	M	mature	08/04/21	Scansano (GR)	1.25 × 10^4^
	#4	M	yearling	26/03/21	Capalbio (GR)	1.06 × 10^4^
	#5	M	yearling	10/04/21	Magliano in Toscana (GR)	2.75 × 10^4^
Fallow deer	#6	M	-	07/11/19	Parco Maremma (GR)	9.3 × 10^3^

**Table 4:** Virological analysis (immunoenzymatic screening and RT-PCR) conducted on liver samples from the 48 roe deer and the 60 fallow deer examined, divided by province origin.

Species	Province	Virological Analysis	
		AgELISA	RT-qPCR
Roe deer	Grosseto	12/35	5/12
	Pisa	4/13	0/4
	Total	16/48	5/16
Fallow deer	Grosseto	3/52	1/3
	Pisa	0/8	-
	Total	3/60	1/3
Total cervids		19/108	6/19

**Table 5:** Results of the immunohistochemical investigation conducted on the six liver samples that scored positive for RT-qPCR. 0 = absence; 1 = slight presence; 2 = moderate presence; 3 = strong presence.

Markers	Histopathological Changes	#1	#2	#3	#4	#5	#6
		Roe Deer					Fallow Deer
Anti-HEV	Positive hepatocytes	1	2	1	1	1	2
	Positive cholangiocytes	3	2	2	2	2	2
Anti-Iba1	Kupffer cells hyperplasia	3	3	3	3	3	3
	Periportal macrophage infiltration	3	3	2	3	2	1
Anti-CD3	Periportal T-cells infiltration	3	3	2	3	3	1
	Sinusoidal T-cells infiltration	3	3	1	2	2	1
Anti-CD20	Periportal B-cells infiltration	1	1	0	1	1	0
	Sinusoidal B-cells infiltration	0	0	0	0	0	0

## Data Availability

The data presented in this study are available on request from the corresponding author.
